# Error in Byline and Table

**DOI:** 10.1001/jamanetworkopen.2026.22341

**Published:** 2026-06-11

In the Research Letter titled “Distance Traveled Among Out-of-State Chicago Abortion Fund Callers,”^1^ published April 24, 2026, there was an error in the listed degree of a coauthor. Dr Daniel’s degree is now listed as a PhD. There were also results transposed in the Table. Results for median distance to provider for hospital and nonhospital level of care have been fixed. This article has been corrected.^1^
